# The Use of Waste Products from the Food Industry to Obtain High Value-Added Products

**DOI:** 10.3390/foods13060847

**Published:** 2024-03-11

**Authors:** Stanisław Kowalski, Dorota Gumul

**Affiliations:** Department of Carbohydrate Technology and Cereal Processing, Faculty of Food Technology, University of Agriculture in Krakow, 122 Balicka Street, 30-149 Krakow, Poland; rrgumul@cyf-kr.edu.pl

Globalization and population expansion are driving the evolution of the food industry, offering an expanded array of food choices to cater to increasingly discerning consumers. This includes those opting for alternative dietary approaches, such as gluten-free diets, even in the absence of celiac disease [1,2,3,4,5]. The processing of both plant and animal raw materials generates a large amount of waste. According to FAO 2019, the global level of waste from animal and meat production is around 13% and from fruit and vegetable processes it is around 22%. According to the report “Wastage footprint: Impacts on natural resources–Summary report” [6], fruit processing, packaging, distribution and consumption generate a huge amount of fruit waste worldwide, approximately 1.81, 6.53 and 32.0 million tons, respectively [7]. In fruit and vegetable processing, an important problem is the management or disposal of waste products, i.e., the parts not used in the technological process, which constitute 10 to 35% of the weight of the processed raw material. However, in the juice production process, the mass of pomace depends on the pressing efficiency. During traditional pressing, the proportion of pomace is 20–25% of the initial weight of the raw material [7,8], and the use of enzyme preparations allows the proportion of pomace to be reduced to approximately 12% of the initial fruit weight. In Europe, by-products from fruit processing account for 8% of all food sector waste [7,8].

Unfortunately, discarded solid and liquid materials are rarely used in the production chain and often serve as animal feed products. Much of this waste remains unprocessed, which causes additional disposal costs for treatment plants and increases the biological burden of wastewater [9,10,11,12]. However, due to growing environmental concerns, intensive research needs to be carried out so that more food waste can be used to add value to new food products. This will consequently lead to maximum benefits for industry, the environment and consumers. Due to the increased awareness of both producers and consumers regarding ecological issues and sustainable development, there is a growing interest in reusing food industry by-products or waste. The number of scientific articles that have been written and published on this topic over the last 30 years shows an exponential increase, which emphasizes its importance and relevance [10,12,13,14,15,16,17,18,19].

Food industry waste can provide a whole range of substances that can improve the nutritional value and functionality of new products. These substances often contain significant amounts of protein, dietary fiber, fat, vitamins, polyphenols, vitamin C and vitamin E, phytosterols, lignans, etc. [13,20]. Examples of waste products that enhance the health-boosting qualities of new products include fruit and vegetable pomace, flour or post-extraction pulp, molasses and other by-products. These products can also be used as substrates for the production of dyes, vitamins or a whole range of other biologically active substances.

In summary, the possibilities of using the entire range of waste products to obtain products with high added value depend on the ingenuity and creativity of both scientists and food producers. We are therefore constantly facing new challenges but also new opportunities to obtain valuable products from waste. This strategy fits perfectly into the current trend of zero-waste technology, which is one of the 17 sustainable development goals included in the United Nations resolution [21].

In the first edition of this Special Issue of *Foods*, “The Use of Waste Products from the Food Industry to Obtain High Value-Added Products”, we presented some developments in and possibilities of the use of these products, a brief summary of which is presented below (Table 1 and Figure 1).

Gumul et al. (2023) analyzed the pulp after the isolation of starch from colored potatoes (*Solanum tubersum* L.) as an ingredient to enrich dessert cookies. It was emphasized that the above-mentioned pulp is a very rich source of polyphenols and fiber. It was found that the pulp from colored potatoes fortified the cookies with polyphenols, flavonoids, anthocyanins and flavonols in amounts that were up to two to four times higher compared to the control cookie. The dessert cookies that contained potato pulp showed up to twice the fiber content and 17% higher protein content, while the fat and ash content remained unchanged compared to the control. Moreover, the cookies with potato pulp were characterized by a 30% lower content of hydroxymethylfurfural and an approximately 40% higher acrylamide content, while showing good physical properties of the final product [SI 1]. In a subsequent publication, Gumul et al. (2023) enriched wheat pasta with commercial dried apple pomace. It was found that the paste containing apple pomace had a four times higher content of polyphenolic compounds and an eight times higher content of flavonoids. Due to the fact that apple pomace is characterized by significant amounts of phenolic acids, quercetin and its derivatives, flavonols and dihydrochalcones, especially the unique phloridzin, this study showed that paste enriched with it also has a high content of these bioactive compounds. Moreover, apple-pomace-fortified paste was characterized by a large amount of dietary fiber, lower protein and fat content, and a higher amount of minerals. It was found that 10% addition guaranteed the quality and beneficial health-promoting properties of the pastes [SI 2]. Both of the above publications take into account the global trend of using zero-waste food technology to expand the range of products enriched with health-promoting compounds derived from waste products.

The aim of the study by Lara-Abia et al. (2023) was to improve the stability and bioavailability of carotenoids obtained from papaya by-products using an oil-in-water (O/W) emulsion. The study investigated the impact of varying concentrations of pectin (1%, 2%, and 3%), a high-molecular emulsifier, combined with Tween 20, a low-molecular emulsifier, under different homogenization conditions. The aim was to identify the optimal parameters for creating stable oil-in-water (O/W) emulsions with encapsulated carotenoids. Three oils were used to formulate these O/W emulsions: soybean, sunflower and coconut. The microstructure (confocal and optical microscopy) of the O/W carotenoid emulsions and their behavior during in vitro digestion phases were investigated. Additionally, the bioavailability of select individual encapsulated papaya carotenoids was tested. It was found that O/W carotenoid emulsions from sunflower oil had a smaller average particle size, higher negative ζ potential and higher viscosity than O/W emulsions from soybeans. Particle size reduction in O/W emulsions using the HPH process improved the bioavailability of papaya-encapsulated carotenoids. In these O/W emulsions, depending on the vegetable oil, the carotenoid with the highest bioavailability was lycopene (71–64%), and that with the lowest bioavailability was (all-E)-β-cryptoxanthin laurate (7–4%). Analogous properties were demonstrated by O/W carotenoid emulsions with soybean oil. It was found that the O/W coconut oil microemulsion was not stable. It has been shown that the high degree of unsaturation of fatty acids in sunflower oil and soybean oil can promote the formation of small droplets and increase the elasticity of the interfacial surface in emulsions due to unsaturated bonds. Studies have proven that microencapsulated carotenoids from papaya by-products have better bioavailability compared to non-microencapsulated products due to the protective effect [SI 3].

Grzelczyk et al. (2003) used by-products generated during olive oil production to prepare biodegradable packaging materials. The authors used 70–80% olive oil, flour/groats and lecithins to produce biodegradable products. Then, they baked these dishes for an hour and a half at 180 degrees Celsius and then covered them with beeswax and heated them again. The basic mechanical and physical parameters of these vessels were examined, and a thermal analysis of the final products was performed. The analysis encompassed an examination of the color and functional characteristics of these containers, alongside assessments of their biodegradability. Furthermore, the utilization of waste products with significant health-promoting benefits prompted investigations into antioxidant activity and total polyphenol content. The authors concluded that it is possible to create disposable packaging from natural sources using olive oil by-products, which can easily replace plastic packaging because they have good functional mechanical and physical properties. A storage test of the produced disposable packaging clearly proved that time has no impact on the properties of the product [SI 4].

Qin et al. (2003) analyzed the biotechnological aspect of using Jiuzao for the production of xylooligosaccharides. These authors investigated the preparation of xylooligosaccharides (XOSs) from Jiuzao through autohydrolysis combined with enzymatic hydrolysis using a thermostable xylanase. It was found that XOSs from Jiuzao could be produced using pre-autohydrolysis and enzymatic hydrolysis with a thermostable xylanase. It was shown that treating Jiuzao at a temperature of 181.5 °C for 20 min at a solid-to-liquid ratio of 1:13.6 enabled efficient hydrolysis with thermostable xylanase to produce XOSs. After enzymatic optimization of the hydrolysis conditions, the highest yield of XOSs from Jiuzao was 34.2% at 60 °C and pH 5 with 15 U/mL XynAR for 2 h. This process holds great promise for the practical production of XOSs from Jiuzao [SI 5].

One of the more widely discussed themes in this Special Issue was the use of spent coffee grounds after the coffee brewing process. This issue was described in two articles by Ibtissam Bouhzam and coauthors [SI 6, SI 7]. In their work, they focused on determining the efficiency of the extraction of polyphenolic compounds, especially chlorogenic acid and caffeine, using water–ethanol and water–acetone systems from coffee grounds. In their work, the authors took into account factors such as the method of drying coffee grounds and storage time on the extraction efficiency of individual biologically active compounds. They also developed quick and effective methods for extracting the above-mentioned compounds using supramolecular solvents and water (mechanical mixing method and ultrasonic extraction support). The results indicated that both the type of solvent and the method of extraction had a significant influence on the amount of extracted biologically active substances.

The next group of articles published as part of this Special Issue were works on the use of by-products from date processing. This topic was studied by Alqahtani et al., and the results were described in two research papers. In the first work [SI 8], the by-products of date fruit seed (DFS) were used to increase the amount of fiber in processed cheese. DFS was used in the range from 0 to 20% and its addition was increased by 5%. The authors observed quality changes in the obtained product, in particular changes in the texture, microstructure, shelf life and sensory characteristics. As the proportion of DFS increased, the structure of the cheese became less compact and the color became darker. The authors suggested that the acceptance of such a fortified product should be achieved up to an additive amount of 10% [SI 8].

In the next work [SI 9], the authors focused on the possibility of using the remains of pressing date syrup. A by-product known as date press cake (DPC) was used to increase the functionality of yogurt. DPC (2, 4 and 6%) was used before fermentation, and the products were tested for changes in pH, acidity, syneresis, water holding capacity, viscosity, and color, among others. The changes in individual parameters were described using appropriate mathematical models. It was found, among other things, that the addition of DPC reduces syneresis and increases the viscosity of yogurt. The best sensory acceptance was found in yogurt with 2% DPC [SI 9].

Ciesarová et al. described the use of asparaginase to reduce the formation of acrylamide in biscuits with the addition of sea buckthorn berries. The sea buckthorn pomace used in this research was a by-product of juice production. Since this pomace is rich in biologically active substances, its reuse is justified. However, due to the high content of the amino acid asparagine in sea buckthorn, this promising material contributes to the undesirable formation of acrylamide. The use of an enzymatic treatment allowed the authors to reduce the asparagine content from 1834 mg/kg to 89 mg/kg and reduce the formation of acrylamide from 35% to 64%, depending on the type of flour from which the biscuits were baked [SI 10].

## Figures and Tables

**Figure 1:**
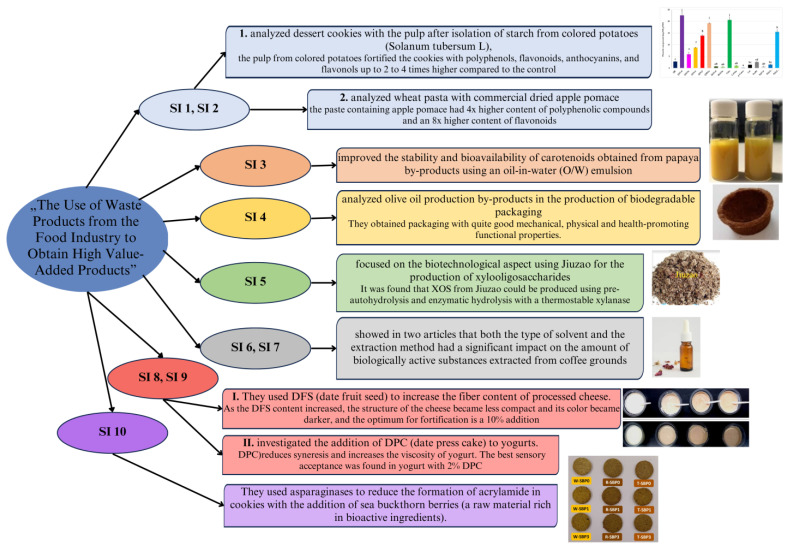
Main directions of research presented in the Special Issue.

**Table 1:** List of articles published in the 1st edition of this Special Issue of *Foods*, “The Use of Waste Products from the Food Industry to Obtain High Value-Added Products”.

Article Symbol	Bibliography	By-Product Used
SI 1	Gumul, D.; Ziobro, R.; Korus, J.; Surma, M. Pulp from Colored Potatoes (*Solanum tuberosum* L.) as an Ingredient Enriching Dessert Cookies. *Foods* **2023**, *12*, 3735.	Potato pulp
SI 2	Gumul, D.; Kruczek, M.; Ivanišová, E.; Słupski, J.; Kowalski, S. Apple Pomace as an Ingredient Enriching Wheat Pasta with Health-Promoting Compounds. *Foods* **2023**, *12*, 804.	Apple pomace
SI 3	Lara-Abia, S.; Lobo, G.; Pérez-Pascual, N.; Welti-Chanes, J.; Cano, M.P. Improvement in the Stability and Bioaccessibility of Carotenoid and Carotenoid Esters from a Papaya By-Product Using O/W Emulsions. *Foods* **2023**, *12*, 2654	Papaya pomace
SI 4	Grzelczyk, J.; Oracz, J.; Gałąazka-Czarnecka, I. Quality Assessment of Waste from Olive Oil Production and Design of Biodegradable Packaging. *Foods* **2022**, *11*, 3776.	Olive oil pomace
SI 5	Qin, L.; Ma, J.; Tian, H.; Ma, Y.; Wu, Q.; Cheng, S.; Fan, G. Production of Xylooligosaccharides from Jiuzao by Autohydrolysis Coupled with Enzymatic Hydrolysis Using a Thermostable Xylanase. *Foods* **2022**, *11*, 2663.	Jiuzao (a mixture of grain and rice hull residues after solid-state fermentation)
SI 6	Bouhzam, I.; Cantero, R.; Margallo, M.; Aldaco, R.; Bala, A.; Fullana-i-Palmer, P.; Puig, R. Extraction of Bioactive Compounds from Spent Coffee Grounds Using Ethanol and Acetone Aqueous Solutions. *Foods* **2023**, *12*, 4400.	Spent coffee Grounds
SI 7	Bouhzam, I.; Cantero, R.; Balcells, M.; Margallo, M.; Aldaco, R.; Bala, A.; Fullana-i-Palmer, P.; Puig, R. Environmental and Yield Comparison of Quick Extraction Methods for Caffeine and Chlorogenic Acid from Spent Coffee Grounds. *Foods* **2023**, *12*, 779.	Spent coffee Grounds
SI 8	Alqahtani, N.K.; Alnemr, T.M.; Alqattan, A.M.; Aleid, S.M.; Habib, H.M. Physicochemical and Sensory Properties and Shelf Life of Block-Type Processed Cheeses Fortified with Date Seeds (*Phoenix dactylifera* L.) as a Functional Food. *Foods* **2023**, *12*, 679.	Date fruit seeds
SI 9	Alqahtani, N.K.; Alnemr, T.M.; Alsalem, A.K.; Alotaibi, M.M.; Mohammed, M. Experimental Investigation and Modeling for the Influence of Adding Date Press Cake on Drinkable Yogurt Quality. *Foods* **2023**, *12*, 1219.	Date press cake
SI 10	Ciesarová, Z.; Kukurová, K.; Jelemenská, V.; Horváthová, J.; Kubincová, J.; Belović, M.; Torbica, A. Asparaginase Treatment of Sea Buckthorn Berries as an Effective Tool for Acrylamide Reduction in Nutritionally Enriched Wholegrain Wheat, Rye and Triticale Biscuits. *Foods* **2023**, *12*, 3170.	Sea buckthorn berry pomace

## Data Availability

No new data were created or analyzed in this study. Data sharing is not applicable to this article.

## References

[1] Leonard M.M., Sapone A., Catassi C., Fasano A. (2017). Celiac Disease and Nonceliac Gluten Sensitivity. A Review. JAMA.

[2] Kruczek B., Gumul D., Olech E., Gambuś H. (2017). Diet and the Context of Fruit Industry. Econ. Environ. Stud..

[3] Gómez M., Martinez M.M. (2018). Fruit and vegetable by-products as novel ingredients to improve the nutritional quality of baked goods. Crit. Rev. Food Sci. Nutr..

[4] Padayachee A., Day L., Howell K., Gidley M.J. (2017). Complexity and health functionality of plant cell wall fibers from fruits and vegetables. Crit. Rev. Food Sci. Nutr..

[5] Makovicky P., Makovicky P., Caja F., Rimarova K., Samasca G., Vannucci L. (2020). Celiac disease and gluten-free diet: Past, present and future. Gastroenterol. Hepatol. Bed Bench.

[6] FAO (2019). The State of Food and Agriculture 2019. Moving Forward on Food Loss and Waste Reduction.

[7] Srivastava N., Srivastava M., Alhazmi A., Kausar T., Haque S., Singh R., Ramteke P.W., Mishra P.K., Tuohy M., Leitgeb M. (2021). Technological advances for improving fungal cellulase production from fruit wastes for bioenergy application: A review. Environ. Pollut..

[8] Kawecka L., Galus S. (2021). Wytłoki owocowe—Charakterystyka i możliwości zagospodarowania [Fruit pomace—Characteristics and possibilities of recycling]. Postępy Tech. Przetwórstwa Spożywczego.

[9] Sadh P.K., Duhan S., Duhan J.S. (2018). Agro-industrial Wastes and Their Utilization Using Solid State Fermentation: A Review. Bioresour. Bioproc..

[10] Reguengo L.M., Salgaço M.K., Sivieri K., Maróstica M.R.J. (2022). Agro-industrial by-products: Valuable sources of bioactive compounds. Food Res. Int..

[11] Lemes A.C., Egea M.B., Oliveira Filho J.G., Gautério G.V., Ribeiro B.D., Coelho M.A.Z. (2022). Biological Approaches for Extraction of Bioactive Compounds from Agroindustrial By-products: A Review. Front. Bioeng. Biotechnol..

[12] Santos D., da Silva J.A.L., Pintado M. (2022). Fruit and Vegetable By-Products’ Flours as Ingredients: A Review on Production Process, Health Benefits and Technological Functionalities. LWT.

[13] Parveen H., Bajpai A., Bhatia S., Singh S. (2017). Analysis of biscuits enriched with fibre by incorporating carrot and beetroot pomace powder. Indian. J. Nutr. Diet..

[14] Kultys E., Moczkowska-Wyrwisz M. (2022). Effect of using carrot pomace and beetroot-apple pomace on physicochemical and sensory properties of pasta. LWT.

[15] Gumul D., Ziobro R., Korus J., Kruczek M. (2021). Apple pomace as a source of bioactive polyphenol compounds in gluten-free breads. Antioxidants.

[16] Maner S., Sharma A.K., Banerjee K. (2015). Wheat flour replacement by wine grape pomace powder positively affects physical, functional and sensory properties of cookies. Proc. Natl. Acad. Sci. India Sect. B Biol. Sci..

[17] Padalino L., Conte A., Lecce L., Likyova D., Sicari V., Pellicanò T.M., Poiana M., Del Nobile M.A. (2017). Functional Pasta with Tomato By-Product as a Source of Antioxidant Compounds and Dietary Fibre. Czech J. Food Sci..

[18] Xu J., Bock J.E., Stone D. (2020). Quality and Textural Analysis of Noodles Enriched with Apple Pomace. J. Food Process. Preserv..

[19] Tolve R., Pasini G., Vignale F., Favati F., Simonato B. (2020). Effect of Grape Pomace Addition on the Technological, Sensory, and Nutritional Properties of Durum Wheat Pasta. Foods.

[20] Sagar N.A., Pareek S., Sharma S., Yahia E.M., Lobo M.G. (2018). Fruit and vegetable waste: Bioactive compounds, their extraction, and possible utilization. Compr. Rev. Food Sci. Food Saf..

[21] United Nations (2015). Resolution Adopted by the General Assembly on 25 September 2015, 526 A/RES/70/1, Transforming our World: The 2030 Agenda for Sustainable Development, 527, United Nations. https://undocs.org/en/A/RES/70/1.

